# Supplementary material for: Early evolution of small body size in Homo floresiensis
Source: Nat Commun. 2024 Aug 6;15:6381. doi: 10.1038/s41467-024-50649-7 (PMC11303730; doi:10.1038/s41467-024-50649-7)
Supplement: Supplementary file 1 — Supplementary Information [file 41467_2024_50649_MOESM1_ESM.pdf]

Supplementary Information for

Early evolution of small body size in *Homo floresiensis*

Yousuke Kaifu, Iwan Kurniawan, Soichiro Mizushima, Junmei Sawada, Michael Lague, Ruly Setiawan, Indra Sutisna, Unggul P. Wibowo, Gen Suwa, Reiko T. Kono, Tomohiko Sasaki, Adam Brumm, Gerrit D. van den Bergh

This PDF file includes:

Supplementary Notes 1 to 7

Supplementary Figures 1 to 5

Supplementary Tables 1 and 2

Supplementary References

Supplementary Note 1:
Stratigraphic and taphonomic context of the hominin fossils

Between 2013 and 2017, excavations in the Upper Fossil-bearing interval of Mata Menge covered a surface area of 210 m^2. The exposed sequence consists of three distinct sedimentary facies types, which are, starting from the lowest, as follows:

1) Layer III is a minimum 30cm-thick medium to coarse-grained sandstone of fluvial origin, overprinted by a reddish brown paleosol. The top constitutes an undulous erosive surface, whereas the base has not been reached in any of the excavations. This layer must have been well-consolidated when its top was eroded, because the undulating top surface locally exhibits steep-sided rounded cliffs[1]. Scarce fossil vertebrate remains do occur in this unit, although in a poor state of preservation, and in much lower concentrations than in the overlying Layer II.

2) Layer II consists of grey, poorly sorted medium to fine sand, locally fining-upwards to very fine sand. This layer has a maximum thickness of 50 cm, tapers out over a horizontal distance of 5-7 m towards the west, and abuts against the elevated relief areas of the underlying Layer III. Layer II filled in a channel that was eroded in the already hardened substrate of the reddish brown palaeosol of Layer III. The upper boundary of Layer II is plane to slightly undulous (Fig. 2). Subangular to subrounded volcanic pebbles with a maximum diameter of 90 mm (average of 10 largest clasts) are scattered at the base of Layer II where it is thickest, with fewer smaller isolated pebbles occurring in the lower half of the layer. Rounded rip clasts of fine-grained muddy sediment are concentrated in certain intervals, but otherwise Layer II appears massive except for locally preserved faint laminations and early diagenetic Liesegang bands. Layer II contains moulds of freshwater gastropods, grassy leaf fossils, and vertebrate remains. Scattered stone artefacts also occur dispersed in Layer II[1].

3) A 6.5 m-thick series of massive, tuffaceous clay-rich mudflow layers of variable thickness (Layers Ia-f) covers Layers II and III. The lowest three of these mudflows (Layers Id-f) have a limited thickness of less than 10 cm and are only developed in the western excavation area where they directly overlie the irregular top surface of Layer III unconformably. Towards the southeast, the mudflow Layer I-c directly overlies Layer II (Fig. 2). The boundary between the top of sandstone Layer II and the base of mudflow Layer I-c can be blurry but is relatively sharp in other places. Up to 20 cm of the basal part of the lowest mudflow that overlies sandstone Layer II contains rounded muddy rip-up clasts and sandy material derived from the

underlying sandy layer, as well as small fossils and fossil fragments, but higher up these coarser components rapidly decrease in abundance (see Fig. 2e). Fragmented freshwater diatoms are common in the mudflows.

Vertebrate fossils are widely represented in Layer II, with over 15,500 fossil vertebrate specimens excavated between 2013-2019[2]. Fossils identifiable as *Stegodon* comprise 34% of the assemblage. In addition, unidentifiable bone fragments (43% of the total assemblage, probably mostly representing *Stegodon* bone fragments[3]), indicate a high fragmentation of the skeletal material. Murine rodent fossils comprise 19% of the assemblage, while the remainder of taxa represented (crocodiles, varanids, birds, frogs) each occupy less than 2% of the total. The lowest proportion is represented by hominin fossils (0.06%).

There is evidence for fluvial transportation of many of the vertebrate fossils prior to burial, with most specimens exhibiting some degree of fracturing. Apart from some excavation damage, old dry bone fractures with ragged surfaces are prevalent. Many bone fragments are weathered to some degree, while other bone and molar fragments tend to be rounded by fluvial transport [3]. However, complete skeletal elements, molars and tusks of *Stegodon* are also common. Some of the heavier *Stegodon* elements, including large fragments of long bones or tusks, occur at the base of Layer II, and in some places are surrounded by pebble lag concentrations. Other fossil specimens are present in the middle of Layer II. Some large *Stegodon* bones were recovered lying on top of Layer II and surrounded by mudflow sediment (see Fig. 2c). Generally, the *Stegodon* bones are not articulated, except for one instance where a single concentration of four ribs and three thoracic vertebrae were found articulated in Layer II (in quadrant P22; refer Fig. 2g for the excavation plan grid). Matching isolated *Stegodon* molars from the left and right side of single individuals have been found from 2 to10 m apart, and in addition the left and right pelvis shown in Figure 2c, lying on top of Layer II, also belonged to one *Stegodon* individual. Skeletal elements and teeth of smaller taxa occur throughout Layer II, notably isolated crocodile and Komodo dragon teeth, and murine rodent postcranial elements, jaws, and isolated incisors and molars. Small fossils of vertebrates are also incorporated in the basal ~20 cm of the lowest mudflow layer.

These combined observations suggest that most of the vertebrate fossils were considerably fragmented and water-transported prior to burial. Some of the broken larger bones may have been fractured due to trampling by *Stegodon*, whereas water transport also may have contributed to the fragmentation, followed by rounding during the water transport[3]. Variable weathering stages (not yet studied systematically) indicate that at least some of the bones were exposed on the surface for a considerable period of time prior to transport and burial[3]. Only in one instance (the articulated vertebrae and

rib concentration of a single *Stegodon* individual), burial must have taken place not too long following the death of that individual.

The fossil assemblage associated with Layer II thus seems to represent a mixture of bones that accumulated over a longer period of time in a small streambed, and more complete fossils that accumulated in the same stream bed closer to the moment that the exposed bedding of the stream was sealed off by the sequence of mudflows. However, the vast majority of the animal carcasses were exposed on the surface for long enough to allow loss of soft tissues and disarticulation. Crocodiles and Komodo dragons have also probably contributed to the disarticulation of the *Stegodon* carcasses. The fining upward sequence of Layer II suggests that the large disarticulated *Stegodon* bones lying on top of Layer II accumulated in the river bedding, after a waning stage of waterflow had deposited the very fine sandstone at the top of Layer II, otherwise the sandy sediment would have at least partly covered these bones.

The fine-grained mudflow sediment that covered the stream bedding is thought to have originated from the Welas Caldera lake some four km to the northwest of Mata Menge, presumably by overflow of the lake during the volcanic activity of one of the volcanic cones situated inside the caldera [2]. The first mudflows arriving at the Mata Menge site eroded and incorporated some sandy material and small bone fragments from the streambed, but appear to have rapidly lost erosive strength as the large *Stegodon* bones on top of Layer II do not seem to have moved far, as evidenced by the two pelvis fragments belonging to the same individual.

The ten isolated hominin fossils are all from a narrow east-west extending area that runs parallel to the paleochannel axis (Fig. 2g; see also Extended Data Fig. 2 in ref. 1). These ten fossils, mostly consisting of dental elements, represent at least four individuals (see Discussion), suggesting that the Mata Menge hominin assemblage, like the other fauna, was highly scattered and dispersed. The concentration of these hominin fossils in the upper 10 cm of Layer II, and their overall good preservation, also suggests limited reworking and a narrower time frame for the accumulation compared to the more evenly distributed other taxa throughout Layer II. Incorporation of the hominin fossils into the upper 15 cm of Layer II must have occurred relatively close to the moment that Layer II was buried by the Layer I mudflows. The *Stegodon* bones lying on top of Layer II may have reached their final destination around the same time. Regarding the cause of death of the hominin and *Stegodon* individuals of which fossils had accumulated in the upper part or on top of Layer II, it is unlikely that this was related to volcanic activity inside the caldera lake that generated the mudflows, because articulated skeletons or parts of skeletons would be prevalent on the surface of Layer II, which is not the case.

Supplementary Note 2:
Morphological description of SOA-MM9

SOA-MM9 is a distal shaft of the right humerus which measures 88 mm in maximum preserved length (Fig. 1). The specimen was damaged in the process of excavating it from the extremely hard sandstone of Layer II. It was recognized as a hominin humerus after reconstruction in laboratory. No taphonomic deformation is evident, and the bone surfaces are intact except for microscopic damages. All the recovered fragments could be refitted perfectly, except for several pieces at the posterior surface and the proximal end which had been lost in the field. A small fragment on the posterior surface is slightly misaligned in the reconstruction (indicated by the 'star' in Fig. 5), but this does not affect the overall morphology of the shaft. The distal fracture occurred before or during the fossilization immediately proximal to the missing epiphysis. Proximally, the excavation damage occurred at the mid-shaft level. The distal shaft of SOA-MM9 is straight. Viewed anteriorly, it is nearly symmetrical with modest flare of the medial and lateral supracondylar crests. The prominent anterior ridge emerges from the center of the distal end and continues proximally with a slight lateral inclination to continue to the deltopectoral crest.

Supplementary Note 3:
Additional notes on the length estimation of SOA-MM9

Our estimation that the SOA-MM9's proximal end is very close to the original 50% level is also consistent with the following observation. As illustrated in Supplementary Fig. 1, a hominin right humerus typically shows an ovoid mid-shaft cross-section with its long axis more or less twisted clockwise when viewed proximally (i.e., the anterior border is located laterally to the midline of the shaft). Distally, the anterior border shifts its relative position medially, and the cross-section becomes a rounded triangle, before assuming a flattened distal contour. Near the distal end, the shaft curves more or less anteriorly, the medial and lateral supracondylar crests develop, and the cortical bone becomes thin. The same transformation is evident in the serial slices and profiles of SOA-MM9 (Fig. 5), and a part of these aspects are numerically confirmed as the changes in the ratio of maximum to minimum second moments of area (Imax/Imin_tcs, which reflects the flatness), weak axis angle (WA angle, which reflects the orientation of the long axis), and relative cortical bone thickness (CA/TA) shown in Supplementary Fig. 2.

To estimate the original vertical level of the hOF point on the SOA-MM9 humerus, we referred to the two shorter humeral subsamples from our modern human sample (N = 366, including modern and prehistoric Japanese and modern Indians) as described in

the main text. This was because the vertical level of the hOF point, i.e., the ratio of the distal epiphysis length (projected length from the hOF to the distal end) relative to the maximum humeral length, is not constant across humeri with varying length. In our modern human sample, this ratio was negatively correlated with the maximum humeral length in both male ($N = 155$, $r = -0.343$, $p < 0.00001$) and female ($N = 121$, $r = -0.306$, $p < 0.001$) samples.

Supplementary Note 4:
Note on the molar crown shape analysis

Recently, Zanolli and colleagues claimed that, based on morphometric analyses of molars, some dental specimens from the Sangiran Lower assemblage (e.g., Sangiran 5, Sangiran 6a and FS-77) are not hominins but belonged to an unknown ape species[4]. This suggestion is relevant to the present study because our early Javanese *H. erectus* M_3 sample includes FS-77. The study by Zanolli also implies that not only these specimens but also other robust M_3s included in our sample, such as Sangiran 6b, are also non-hominins. We do not agree with this conclusion, because the mandible of Sangiran 5 and Sangiran 6a are clearly those of *Homo*[5], and their study did not include early African *Homo* sample, which shows considerable affinities with the earliest Sangiran dentognathic remains[5,6]. Irrespective of this debate, FS-77 and Sangiran 6a exhibit very similar crown form with the other Sangiran *H. erectus* teeth (Fig. 3d). Because of this, the inclusion or exclusion of these teeth do not significantly affect our conclusion about the affinities of SOA-MM11.

Supplementary Note 5:
Variation in body size of Liang Bua *H. floresiensis*

It should be emphasized that LB1 may be a relatively large-sized individual among the Liang Bua *H. floresiensis* so far discovered[7]. For example, the LB8/1 tibia is 8–10% shorter than that of LB1 (LB1/13)[7]. The capitate (LB20) and hamate (LB21/22) from different individuals are smaller than those of LB1 (LB1/45 and LB1/46) by 1–14%[8]. Still, these ratios as compared to those between the Mata Menge hominins and LB1 (Table 2) suggest that the individuals from Mata Menge were comparable to the smallest Liang Bua individuals in terms of the available mandibular, dental, and humeral measurements.

Supplementary Note 6:
Allometric relationship of molar crown shape

Mata Menge *H. floresiensis* displays early Javanese *H. erectus*-like molar crown forms as demonstrated previously for $M_{1/2}$[9] and by this study for M_3, but is substantially smaller in molar crown sizes compared to *H. habilis s.l.* If the latter is ancestral to *H. floresiensis* as suggested by some researchers[10-12], *H. habilis s.l.* molars may have an allometric trend in which a smaller tooth tends to exhibit *H. erectus*-like crown form. This trend should be observed as negative correlation between molar crown size and PC1 score for all molars (Fig. 3 in Ref. 9 and Fig. 3 of this study). However, contrary to these expectations, correlation coefficients calculated between the square root of computed crown area (mesiodistal × buccolingual crown diameters) and the PC1 score are 0.32 for M_1 ($N = 11$), 0.18 for M_2 ($N = 9$), and 0.23 for M_3 ($N = 13$).

Supplementary Note 7:
Additional notes on the humeral comparative samples

Two of the *H. naledi* specimens (U.W. 101-466, U.W. 101-1240) present an unusually projecting lateral supracondylar ridge that strongly influences the profile of the lateral aspect of the section (especially in the case of U.W. 101-1240). A highly projecting ridge requires digitization via many landmarks that would otherwise be used to define the main diaphysis. The variation associated with these ridge landmarks ends up redistributed to the other landmarks during Procrustes superimposition with other specimens. Consequently, the lateral ridge has an undesirably strong influence on the way that these two *H. naledi* specimens align with the other fossils (i.e., it produces greater dissimilarity to other specimens than one would expect based on the shape of the main diaphyseal outline). To mitigate this effect, the lateral supracondylar ridge of the above two specimens was digitally removed and landmarks were placed along a reconstructed lateral border. Since the lateral supracondylar ridge on these specimens is relatively narrow (anteroposteriorly), minimal reconstruction of the lateral border is necessary once the ridge is virtually removed. Ignoring the projecting lateral supracondylar ridge on U.W. 101-466 and U.W. 101-1240 results in much greater morphological homogeneity among the five *H. naledi* specimens. The average Procrustes distance to the three unreconstructed *H. naledi* specimens decreases from 0.173 to 0.054 in the case of U.W. 101-1240 and from 0.104 to 0.047 in the case of U.W. 101-466. Moreover, the Procrustes distance between U.W. 101-1240 and U.W. 101-466 drops from 0.085 (unreconstructed) to 0.039 (reconstructed). While the highly projecting nature of the lateral supracondylar ridge of U.W. 101-466 and U.W. 101-1240 should certainly be acknowledged in comparative studies, the virtual “removal” of

the ridge provides for a more reasonable set of shape comparisons with respect to the particular methodology of the present study.

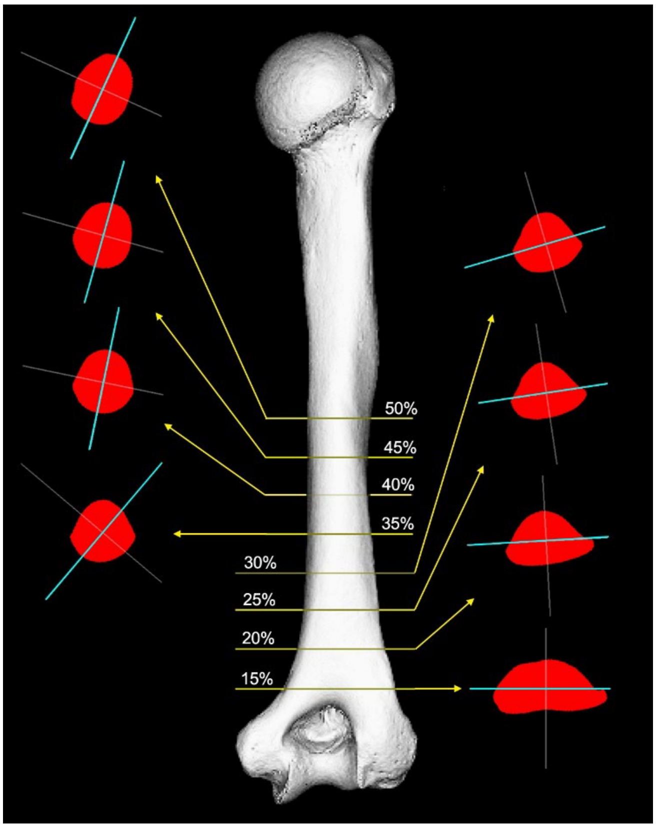

Supplementary Fig. 1. An example of the CT sections prepared to measure the cross-sectional properties. Strong axis (the white line) and weak axis (the blue line) are indicated for each section. The weak axis at 15% level is used as the x-axis to measure the WA (weak axis) angle. The specimen shown here is a short Jomon humerus (UMUT131365) with the maximum length of 242 mm.

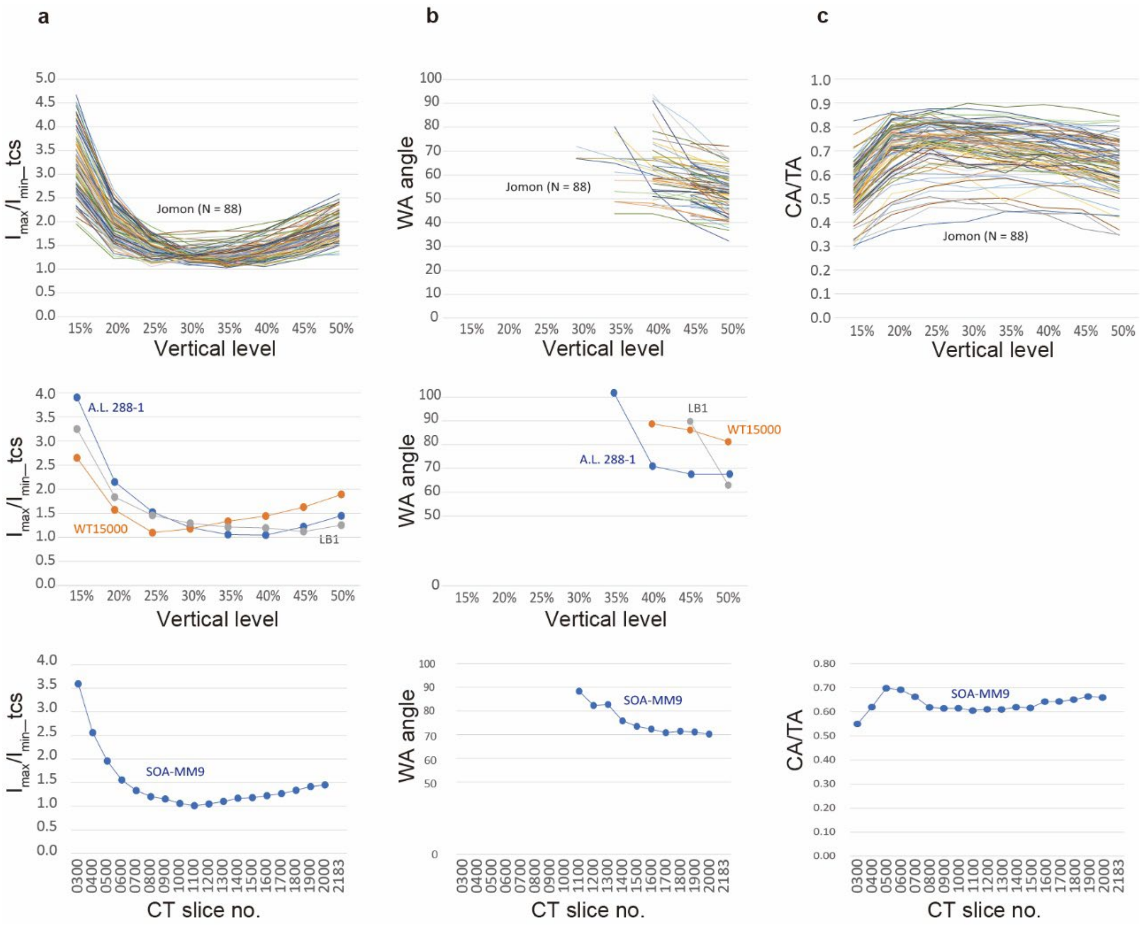

Supplementary Fig. 2. Cross-sectional properties in extant and fossil hominin distal humeri. a, Ratio between the maximum and minimum second moment of area based on the total cross-sectional surface (I_{max}/I_{min}_tcs) as a measure of flatness. This parameter is moderate at the 50% vertical level, decreases and reaches its minimum at the 25−35% levels in 78/88 (89%) of our modern human sample (n=88), and abruptly increases from the 20% to 15% level. **b**, Angle of the weak axis (WA angle) as a measure of the torsion of the distal shaft. The X-axis for this measurement was defined as the line passing through the medial and lateral supracondylar crests (the remotest points to each other) in the 15% section (no. 300 for SOA-MM9). This angle approaches 90º at 30−50% level in our modern human sample (n=88), and then decreases proximally as the anterior ridge shifts laterally. This figure illustrates only that portion to see the mid-shaft morphology. **c**, Ratio between the cortical bone and total sectional area (CA/TA) as a measure of relative cortical bone thickness. This parameter drops substantially from the 20 to 15% level in our modern human sample (n=88), reflecting the cortical bone thinning near the epiphysis. Note that, in all these parameters (**a**-**c**), the fossil specimens show similar trends to the modern human, and SOA-MM9 follows these general patterns if its preserved shaft is assumed to be ~50−13% level. Data for A.L. 288-1 are from the CT sections of the original specimen; KNM-WT 15000 from a cast; LB1 from a 3D print created from CT scan of the original specimen. Only complete humeri are included except for SOA-MM9. Source data are provided as a Source Data file.

a

b

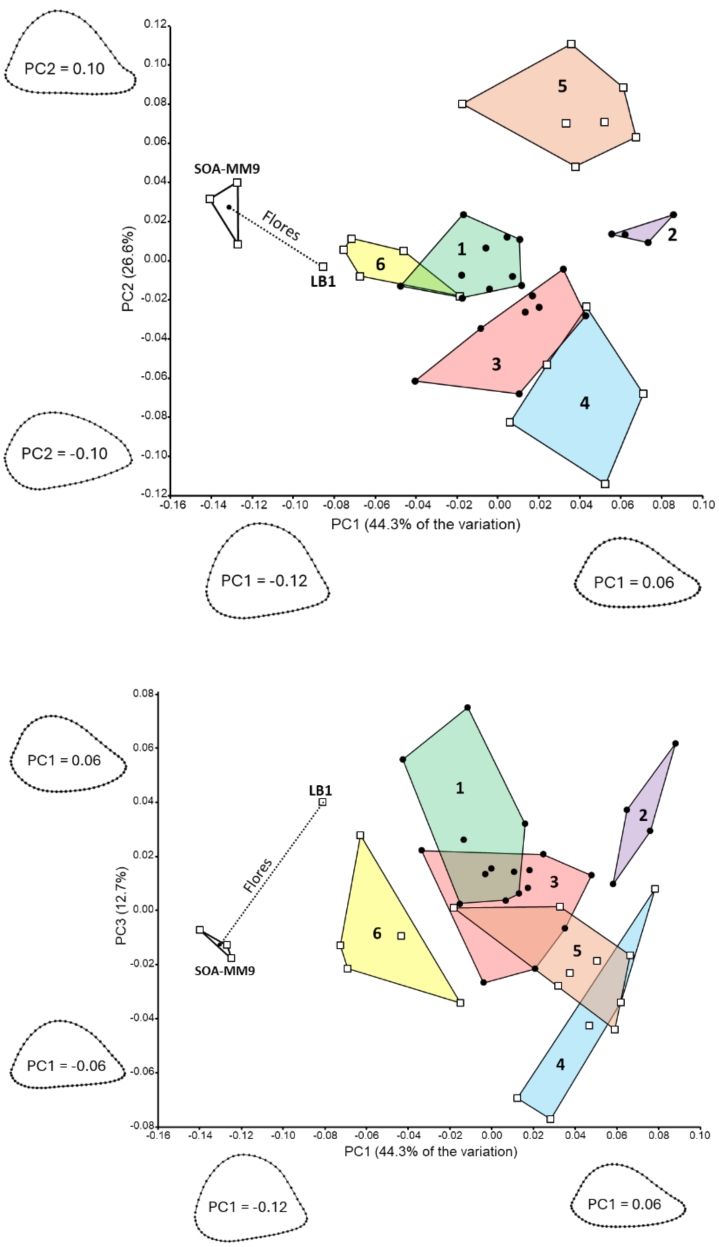

Supplementary Fig. 3. Principal component analysis (PCA) of cross-sectional shape of the distal humeral diaphysis. a, The first two principal components of distal diaphyseal shape among 40 fossil hominin humeri. Convex hulls define the fossil groups (Groups 1-3 = australopith, Group 4 = *H. habilis*, Group 5 = *H. erectus s.l.*, Group 6 = *H. naledi*; see Supplementary Data 3 for more details). The dotted line connects LB1 to the average shape of the three sampled sections of SOA-MM9. Diaphyseal outlines depict shape variation along each component. SOA-MM9 is extreme along PC1 and is most similar in overall shape (based on Procrustes distance) to LB1 and to specimens of *H. naledi* (Group 6). **b**, PC1 and PC3 for the same data. Source data are provided as a Source Data file.

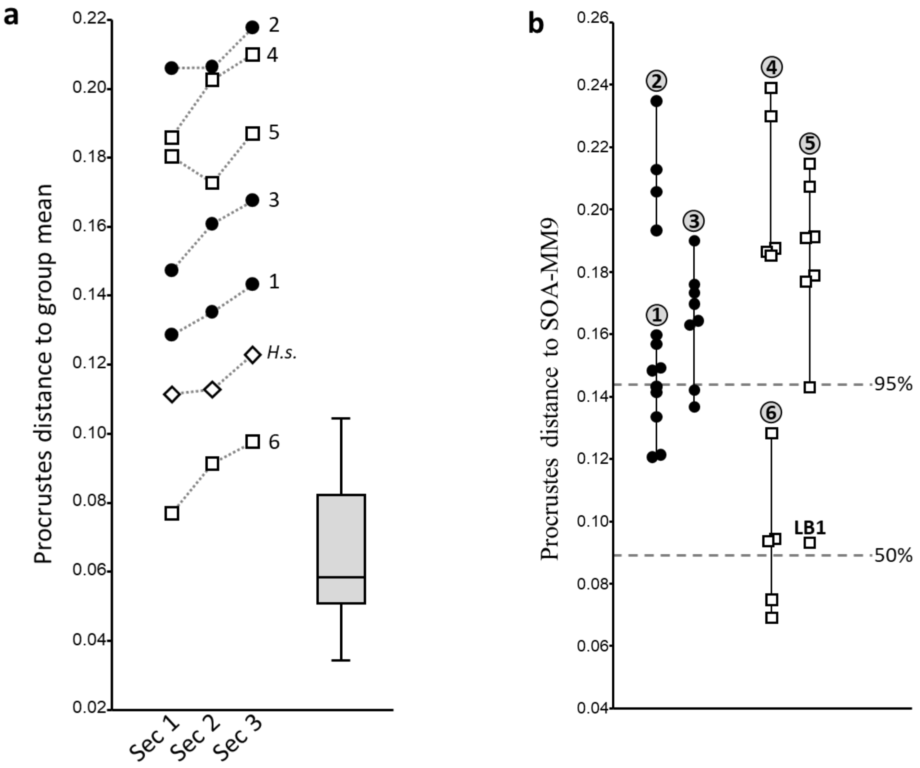

Supplementary Fig. 4. Distal diaphyseal shape similarities as measured by the Procrustes distances. a, Procrustes distances between the distal diaphyseal shape of SOA-MM9 and the group averages for modern humans (*H.s.*) and six groups of fossil humeri (see Supplementary Data 3). Distances are depicted for each of the three sampled sections of SOA-MM9, from distal (Sec 1) to proximal (Sec 3). For comparative context, a standard box plot (five-number summary) shows the distribution of distances between each modern human specimen and the average modern human shape. Among the fossil groups examined here, SOA-MM9 is most similar to Group 6 (*H. naledi*); distances to all other group means fall beyond the modern human comparative distribution. **b**, Procrustes distances between SOA-MM9 (average of three sections) and other fossil hominin humeri with respect to distal diaphyseal cross-sectional shape (black circle = australopith; white square = *Homo*). Comparative morphological groups are numbered as in Supplementary Data 3, which also lists the Procrustes distance to SOA-MM9 for each specimen. The horizontal dashed lines represent the 50th and 95th percentiles for a distribution of all possible pairwise distances (n = 210) within a sample of modern humans (n = 21). SOA-MM9 is most similar to LB1/50 and to specimens of *H. naledi* and does not resemble specimens attributed to early *Homo* (Groups 4 and 5). Source data are provided in Source Data and Supplementary Data 3.

a

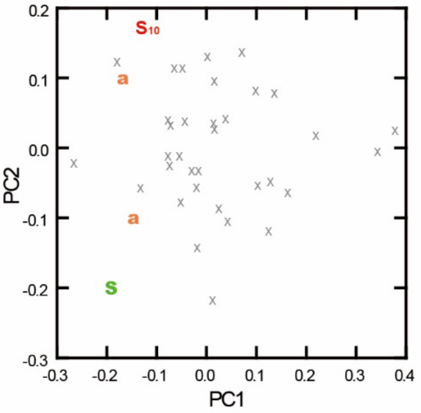

Variable (size-standardized)	PC1	PC2	PC3	PC4
Mesiodistal diameter	-0.691	-0.606	-0.367	0.145
Labiolingual diameter	-0.718	-0.119	0.611	0.312
Crown height	-0.561	0.793	-0.203	0.119
Distal shoulder height	0.962	0.018	-0.101	0.254
Proportion (%)	58	27	10	4
Cumulative proportion (%)	58	85	96	100

a *Au. afarensis*
a *Au. africanus*
h *H. habilis sensu lato*
S *H. erectus* (Sangiran Lower)
S10 SOA-MM10
x *H. sapiens*

b

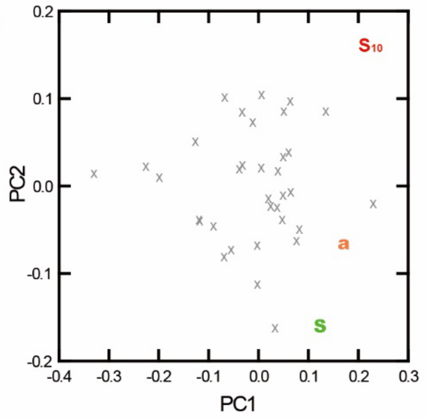

Variable (size-standardized)	PC1	PC2	PC3	PC4	PC5
Mesiodistal diameter	0.752	-0.561	-0.267	-0.220	0.025
Labiolingual diameter	0.774	-0.119	0.087	0.615	0.034
Crown height	0.655	0.739	-0.098	-0.121	0.021
Mesial shoulder height	-0.273	-0.162	0.888	-0.321	0.087
Distal shoulder height	-0.939	0.071	-0.325	0.073	0.047
Proportion (%)	58	23	10	8	0
Cumulative proportion (%)	58	81	91	100	100

Supplementary Fig. 5. Multivariate analyses of maxillary deciduous canine (d^c: SOA-MM10). Results of PCAs based on four (**a**) and five (**b**) linear measurements. PC1s, which explain 57−58% of the total variation, is negatively (a) or positively (b) loaded if the distal shoulder is low relative to the crown length, breadth, and height. Symbols: ‘a’ (orange) = *Au. afarensis*, ‘a’ (red) = *Au. africanus*, ‘h’ (blue) = *H. habilis sensu lato*, ‘S’ (green) = *H. erectus* (Sangiran Lower), ‘s$_{10}$’ (red) = SOA-MM10, ‘x’ = *H. sapiens*. Source data are provided as a Source Data file.

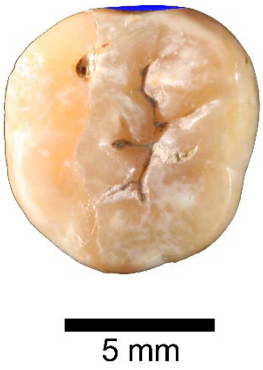

Supplementary Fig. 6. Oocclusal view of the SOA-MM 11 molar crown with reconstruction for mesial interproximal wear. The reconstructed part is indicated in blue.

Supplementary Table 1. Crown dimensions of the new Mata Menge teeth as compared to those of Liang Bua *H. floresiensis*.

Specimen	Tooth	Side	Wear*	Crown diameters						Ref.
				MD † as measured	MD † corrected for wear	BL †	Height buccal crown	Height mesial shoulder	Height distal shoulder	
SOA-MM10	d^c	R	2	4.9	4.9	4.5	6.0 ‡	2.4	2.0 [c]	
SOA-MM11	M_3	L	3	9.0	9.1	8.5	–	–	–	
LB1	M_3	R/L	4/3	8.9/9.8	8.9/9.8	9.5/9.6	–	–	–	49
LB6/1	M_3	R/L	3/3	8.9/8.9	9.0/8.9	8.8/8.5	–	–	–	49

Measurements are in millimeters. *Scored following ref. 50. †Measured following the method of ref. 51. ‡Corrected for wear.

Supplementary Table 2. Comparative dental sample.

Sample	Tooth	Specimens
H. floresiensis (Liang Bua)	Mand. M3	LB1, 6/1
Au. afarensis	Max. dc	LH3/6c*, AL333-66, AL333-104(99)*
Au. africanus	Max. dc	Taung, Stw 151, Stw 305
H. habilis	Max. dc	ER1590
	Mand. M3	KNM-ER 1462*, 1480*, 1801*, 1802*, 1805*, 1812, 3953*, 60000†; OH 4*, 13*, 16*, 27*, 60*; Omo 75-14G*, 75s-16*
Dmanisi *Homo*	Mand. M3	D211‡
H. ergaster	Mand. M3	KNM-ER 730*, 806*, 992*; LH29*; KGA10-1*; SK15
Early Javanese *H. erectus*	Max. dc	S7-83*
(Sangiran Lower)	Mand. M3	Sangiran 1b*, 6b*, 7-77*, 8*, 9*, 22*
Early Javanese *H. erectus* (Sangiran Upper)	Mand. M3	Sangiran 7-23, 7-24*, 21*; Sb8103*
Modern humans	Max. dc	N=62: recent (54) and prehistoric (8: Jomon) Japanese
	Mand. M3	N=225: Global sample from Southeast/Northeast/South Asia, Melanesia, Australia, Africa and Europe

*Specimens used for principal component analyses. †Metric data obtained from ref. 52. ‡Metric data obtained from ref. 53. The other specimens were measured by Y.K. based on the original specimens or high-quality casts. Raw data for the modern human M_3 are available in ref. 54.

Supplementary References

1 Brumm, A. et al. Age and context of the oldest known hominin fossils from Flores. *Nature* 534, 249-253 (2016).

2 van den Bergh, G. D. et al. An integrative geochronological framework for the Pleistocene So'a basin (Flores, Indonesia), and its implications for faunal turnover and hominin arrival. *Quat. Sci. Rev.* 294 107721 (2022).

3 Powley, M. J., Sutisna, I., Mikac, K. M., Wibowo, U. P. & van den Bergh, G. D. The *Stegodon* Bonebed of the Middle Pleistocene Archaeological Site Mata Menge (Flores, Indonesia): Taphonomic Agents in Site Formation. *Quaternary* 4, doi:10.3390/quat4040031 (2021).

4 Zanolli, C. *et al.* Evidence for increased hominid diversity in the Early to Middle Pleistocene of Indonesia. *Nat. Ecol. Evol.* 3, 755-764 (2019).

5 Kaifu, Y. *et al.* Taxonomic affinities and evolutionary history of the Early Pleistocene hominids of Java: dentognathic evidence. *Am. J. Phys. Anthropol.* 128, 709-726 (2005).

6 Tobias, P. V. & Von, K. A comparison between the Olduvai hominines and those of Java and some implications for hominid phylogeny. *Nature* 204, 515-518 (1964).

7 Morwood, M. J. *et al.* Further evidence for small-bodied hominins from the Late Pleistocene of Flores, Indonesia. *Nature* 437, 1012-1017 (2005).

8 Orr, C. M. et al. New wrist bones of *Homo floresiensis* from Liang Bua (Flores, Indonesia). *J. Hum. Evol.* 64, 109-129 (2013)

9 van den Bergh, G. D. *et al. Homo floresiensis*-like fossils from the early Middle Pleistocene of Flores. *Nature* 534, 245-248 (2016).

10 Dembo, M., Matzke, N. J., Mooers, A. O. & Collard, M. Bayesian analysis of a morphological supermatrix sheds light on controversial fossil hominin relationships. *Proc. Biol. Sci.* 282, 20150943 (2015).

11 Dembo, M. *et al.* The evolutionary relationships and age of *Homo naledi*: An assessment using dated Bayesian phylogenetic methods. *J. Hum. Evol.* 97, 17-26 (2016).

12 Argue, D., Groves, C. P., Lee, M. S. Y. & Jungers, W. L. The affinities of *Homo floresiensis* based on phylogenetic analyses of cranial, dental, and postcranial characters. *J. Hum. Evol.* 107, 107-133 (2017).

13 Lovejoy, C. O., Simpson, S. W., White, T. D., Asfaw, B. & Suwa, G. Careful climbing in the Miocene: The forelimbs of *Ardipithecus ramidus* and humans are primitive. *Science* 326, 70-70e8 (2009).

14 Ward, C. V., Kimbel, W. H., Harmon, E. H. & Johanson, D. C. New postcranial fossils of *Australopithecus afarensis* from Hadar, Ethiopia (1990-2007). *J. Hum. Evol.* 63, 1-51 (2012).

15 Feuerriegel, E. M. *et al.* The upper limb of *Homo naledi*. *J. Hum. Evol.* 104, 155-173 (2017).

16 Asfaw, B. *et al. Australopithecus garhi*: a new species of early hominid from Ethiopia. *Science* 284, 629-635 (1999).

17 Ruff, C. B., Burgess, M. L., Ketcham, R. A. & Kappelman, J. Limb Bone Structural Proportions and Locomotor Behavior in A.L. 288-1 ("Lucy"). *PLoS One* 11, e0166095 (2016).

18 Heaton, J. L. *et al.* The long limb bones of the StW 573 *Australopithecus* skeleton from Sterkfontein Member 2: Descriptions and proportions. *J. Hum. Evol.* 133, 167-197 (2019).

19 Churchill, S. E. *et al.* The upper limb of *Australopithecus sediba*. *Science* 340, 1233477 (2013).

20 Churchill, S. E. *et al.* The shoulder, arm, and forearm of *Australopithecus sediba*. *PaleoAnthropology* 2018, 234-281 (2018).

21 Lague, M. R. *et al.* Humeral anatomy of the KNM-ER 47000 upper limb skeleton from Ileret, Kenya: Implications for taxonomic identification. *J. Hum. Evol.* 126, 24-38 (2019).

22 Johanson, D. C. *et al.* New partial skeleton of *Homo habilis* from Olduvai Gorge, Tanzania. *Nature* 327, 205-209 (1987).

23 Haeusler, M. & McHenry, H. M. Body proportions of *Homo habilis* reviewed. *J. Hum. Evol.* 46, 433-465 (2004).

24 Ruff, C. Relative limb strength and locomotion in *Homo habilis*. *Am. J. Phys. Anthropol.* 138, 90-100 (2009).

25 Lordkipanidze, D. *et al.* Postcranial evidence from early *Homo* from Dmanisi, Georgia. *Nature* 449, 305-310 (2007).

26 Ruff, C. Femoral/humeral strength in early African *Homo erectus*. *J. Hum. Evol.* 54, 383-390 (2008).

27 Di Vincenzo, F. *et al.* The massive fossil humerus from the Oldowan horizon of Gombore I, Melka Kunture (Ethiopia, >1.39 Ma). *Quat. Sci. Rev.* 122, 207-221 (2015).

28 Weidenreich, F. The extremity bones of Sinanthropus pekinensis. *Palaeontol. Sin. New Ser. D* 5, 1-82 (1941).

29 Xing, S., Carlson, K. J., Wei, P., He, J. & Liu, W. Morphology and structure of *Homo erectus* humeri from Zhoukoudian, Locality 1. *PeerJ* 6, e4279 (2018).

30 Auerbach, B. M. & Ruff, C. B. Human body mass estimation: a comparison of "morphometric" and "mechanical" methods. *Am. J. Phys. Anthropol.* 125, 331-342 (2004).

31 Ward, C. V., Leakey, M. G. & Walker, A. Morphology of *Australopithecus anamensis* from Kanapoi and Allia Bay, Kenya. *J. Hum. Evol.* 41, 255-368 (2001).

32 Johanson, D. C. *et al.* Morphology of the Pliocene partial hominid skeleton (A.L. 288-1) from the Hadar formation, Ethiopia. *Am. J. Phys. Anthropol.* 57, 403-451 (1982).

33 Lovejoy, C. O., Johanson, D. C. & Coppens, Y. Hominid upper limb bones recovered from the Hadar formation: 1974-1977 collections. *Am. J. Phys. Anthropol.* 57, 637-649 (1982).

34 Toussaint, M., Macho, G. A., Tobias, P. V., Partridge, T. C. & Hughes, A. R. The third partial skeleton of a late Pliocene hominin (Stw 431) from Sterkfontein, South Africa. *S. Afr. J. Sci.* 99, 215-223 (2003).

35 Lague, M. R. & Menter, C. G. in *Hominin Postcranial Remains from Sterkfontein, South Africa* (eds C. V. Ward & B. Zipfel) (Oxford University Press, 2020).

36 Broom, R. Further evidence on the structure of the South African Pleistocene anthropoids. *Nature* 142, 897-899 (1938).

37 Straus, W. L., Jr. The humerus of *Paranthropus robustus*. *Am. J. Phys. Anthropol.* 6, 285-311 (1948).

38 Lague, M. R. & Menter, C. G. DNH 32: A distal humerus of *Paranthropus robustus* from Drimolen, South Africa. *Am. J. Phys. Anthropol.* 162, 255 (2017).

39 Lague, M. R. Taxonomic identification of Lower Pleistocene fossil hominins based on distal

humeral diaphyseal cross-sectional shape. *PeerJ* 3, e1084 (2015).

40 Berger, L. R. *et al. Australopithecus sediba*: a new species of *Homo*-like australopith from South Africa. *Science* 328, 195-204 (2010).

41 Dominguez-Rodrigo, M. *et al.* First partial skeleton of a 1.34-million-year-old *Paranthropus boisei* from Bed II, Olduvai Gorge, Tanzania. *PLoS One* 8, e80347 (2013).

42 Lague, M. R. & Ward, C. in *The Forgotten Lineage(s): Paleobiology of Paranthropus* (eds P. J. Constantino, K. E. Reed, & B. A. Wood) (Springer, Cham, in press).

43 Leakey, R. E. F., Walker, A., Ward, C. V. & Grausz, H. M. in *Hominidae: Proceedings of the 2nd International Congress of Human Paleontology* (ed G. Giacobini) 167-173 (Editoriale Jaca Book, 1989).

44 Lague, M. R. The pattern of hominin postcranial evolution reconsidered in light of size-related shape variation of the distal humerus. *J. Hum. Evol.* 75, 90-109 (2014).

45 Walker, A., Zimmerman, M. R. & Leakey, R. E. A possible case of hypervitaminosis A in *Homo erectus*. *Nature* 296, 248-250 (1982).

46 Lague, M. R., Leakey, M. G., Leakey, L. N. & Jungers, W. L. New hominin fossil humeri from Koobi Fora reflect the diversity of Lower Pleistocene hominins. *PaleoAnthropology* 2016, A17 (2016).

47 Berger, L. R. *et al. Homo naledi*, a new species of the genus *Homo* from the Dinaledi Chamber, South Africa. *Elife* 4, doi:10.7554/eLife.09560 (2015).

48 Brown, P. *et al.* A new small-bodied hominin from the Late Pleistocene of Flores, Indonesia. *Nature* 431, 1055-1061 (2004).

49 Kaifu, Y. *et al.* Descriptions of the dental remains of *Homo floresiensis*. *Anthropol. Sci.* 123, 129-145 (2015).

50 Smith, H. Patterns of molar wear in hunter-gathers and agriculturalists. *Am. J. Phys. Anthropol.* 63, 39-56 (1984).

51 Wood, B. *Koobi Fora Research Project 4: Hominid Cranial Remains*. Vol. 4 (Clarendon Press, Oxford, 1991).

52 Leakey, M. G. *et al.* New fossils from Koobi Fora in northern Kenya confirm taxonomic diversity in early *Homo*. *Nature* 488, 201-204 (2012).

53 Martinón-Torres, M. et al. Dental remains from Dmanisi (Republic of Georgia): morphological analysis and comparative study. *J. Hum. Evol.* 55, 249-273 (2008).

54 Kaifu, Y. et al. Unique dental morphology of *Homo floresiensis* and its evolutionary implications. PLoS ONE 10(11): e0141614 (2015).
