# Supplementary material for: Early evolution of small body size in Homo floresiensis
Source: Nat Commun. 2024 Aug 6;15:6381. doi: 10.1038/s41467-024-50649-7 (PMC11303730; doi:10.1038/s41467-024-50649-7)
Supplement: Supplementary file 3 — Description of Additional Supplementary Files [file 41467_2024_50649_MOESM3_ESM.pdf]

Description of Additional Supplementary Files

Supplementary Data 1: Bone histomorphometric data for the humeral midshafts of SOA-MM9 and comparative modern human sample.

Supplementary Data 2: Humeral metrics for SOA-MM9 and comparative fossil and extant specimens.

Supplementary Data 3: Hominin fossil humeri used in the comparative analysis of distal humeral diaphyseal shape (~19% level).

Supplementary Data 4: Cross-sectional properties of the SOA-MM9 humerus.
