# Supplementary material for: Early evolution of small body size in Homo floresiensis
Source: Nat Commun. 2024 Aug 6;15:6381. doi: 10.1038/s41467-024-50649-7 (PMC11303730; doi:10.1038/s41467-024-50649-7)
Supplement: Supplementary file 5 — Reporting Summary [file 41467_2024_50649_MOESM5_ESM.pdf]

Corresponding author(s): Y. Kaifu, I. Kurniawan, G. van den Bergh

Last updated by author(s): Jun 13, 2024

Reporting Summary

Nature Portfolio wishes to improve the reproducibility of the work that we publish. This form provides structure for consistency and transparency in reporting. For further information on Nature Portfolio policies, see our Editorial Policies and the Editorial Policy Checklist.

Statistics

For all statistical analyses, confirm that the following items are present in the figure legend, table legend, main text, or Methods section.

n/a	Confirmed	
☐	☒	The exact sample size (n) for each experimental group/condition, given as a discrete number and unit of measurement
☐	☒	A statement on whether measurements were taken from distinct samples or whether the same sample was measured repeatedly
☒	☐	The statistical test(s) used AND whether they are one- or two-sided<br>*Only common tests should be described solely by name; describe more complex techniques in the Methods section.*
☒	☐	A description of all covariates tested
☒	☐	A description of any assumptions or corrections, such as tests of normality and adjustment for multiple comparisons
☐	☒	A full description of the statistical parameters including central tendency (e.g. means) or other basic estimates (e.g. regression coefficient) AND variation (e.g. standard deviation) or associated estimates of uncertainty (e.g. confidence intervals)
☒	☐	For null hypothesis testing, the test statistic (e.g. F, t, r) with confidence intervals, effect sizes, degrees of freedom and P value noted<br>*Give P values as exact values whenever suitable.*
☒	☐	For Bayesian analysis, information on the choice of priors and Markov chain Monte Carlo settings
☒	☐	For hierarchical and complex designs, identification of the appropriate level for tests and full reporting of outcomes
☒	☐	Estimates of effect sizes (e.g. Cohen's d, Pearson's r), indicating how they were calculated

Our web collection on statistics for biologists contains articles on many of the points above.

Software and code

Policy information about availability of computer code

Data collection	3D data of SOA-MM9 were acquired from CT scan processed by Analyze 14.0 (AnalyzeDirect, Inc.) and using CT-Rugle 1.2 (Medic Engineering Co.) and Landmark editor software (Wiley et al., 2005. Evolutionary morphing. In: Proceedings of IEEE visualization 2005). Crown contour data of SOA-MM11 was obtained by using Canvas X software (ACD Systems).
Data analysis	Contour data of SOA-MM9 was analyzed by tpsDig2 (Rohlf FJ. 2013. tpsDig, digitize landmarks and outlines. version 2.17. Department of Ecology and Evolution, State University of New York at Stony Brook), tpsRelw software (Rohlf FJ. 2010. tpsRelw, relative warps analysis. version 1.49. Department of Ecology and Evolution, State University of New York at Stony Brook). Contour data for SOA-MM11 was analyzed by SHAPE 1.3 (Iwata and Ukai, 2002).

For manuscripts utilizing custom algorithms or software that are central to the research but not yet described in published literature, software must be made available to editors and reviewers. We strongly encourage code deposition in a community repository (e.g. GitHub). See the Nature Portfolio guidelines for submitting code & software for further information.

Data

Policy information about availability of data

All manuscripts must include a data availability statement. This statement should provide the following information, where applicable:

- Accession codes, unique identifiers, or web links for publicly available datasets
- A description of any restrictions on data availability
- For clinical datasets or third party data, please ensure that the statement adheres to our policy

All data generated or analysed during this study are included in this published article (and its supplementary information files).

Research involving human participants, their data, or biological material

Policy information about studies with human participants or human data. See also policy information about sex, gender (identity/presentation), and sexual orientation and race, ethnicity and racism.

Reporting on sex and gender	n/a
Reporting on race, ethnicity, or other socially relevant groupings	n/a
Population characteristics	n/a
Recruitment	n/a
Ethics oversight	n/a

Note that full information on the approval of the study protocol must also be provided in the manuscript.

Field-specific reporting

Please select the one below that is the best fit for your research. If you are not sure, read the appropriate sections before making your selection.

☐ Life sciences ☐ Behavioural & social sciences ☒ Ecological, evolutionary & environmental sciences

For a reference copy of the document with all sections, see nature.com/documents/nr-reporting-summary-flat.pdf

Ecological, evolutionary & environmental sciences study design

All studies must disclose on these points even when the disclosure is negative.

Study description	Morphological study on hominin fossil bones and teeth discovered by our field research.
Research sample	Hominin fossil bone and teeth collected from Mata Menge, So'a Basin, Flores, Indonesia.
Sampling strategy	Excavations in 2013, 2015, 2016, 2017, 2018, 2019 and 2023.
Data collection	Y,K,, S.M., J.S., M.L. and G.S. collected morphometric data from the reported fossil specimens and comparative materials.
Timing and spatial scale	Morphological data collection started in 2015 and stopped in 2023, when the comparative data set became sufficient to support our conclusions.
Data exclusions	No data were excluded from the analyses.
Reproducibility	Measurement methods are described in the paper.
Randomization	Since the data derive from the three fossils, no randomization was performed
Blinding	No blinding was performed, as this study is based on the obtained fossil sample.

Did the study involve field work? ☒ Yes ☐ No

Field work, collection and transport

Field conditions	Mata Menge is an open archaeological and palaeontological site located in the tropics. Successive fieldworks were conducted between May and November during the dry season, when temperatures range in the upper 20s to lower 30s degrees Celcius. The

	undulating hilly terrain is covered by grassland used for grazing cattle. During excavation shade was created by stretching tarpaulins over the excavation area. Trained personnel from the nearby villages conducted the excavations under supervision of academics. The bone bed from which the fossils were excavated consists of indurated sandstone requiring metal chisels and hammers to be used. This caused inevitable excavation damage to some of the fossils recovered.
Location	The site Mata Menge (-8.691556 S; 121.093997 E) is located in the So'a Basin, which is surrounded by active and inactive volcanoes. The site occurs at an elevation of c 340m above sea level.
Access & import/export	Excavations were conducted with permission from the landowner and permits from the Indonesian Directorate for Higher Education, Research and Technology (RISTEK) and from the Provincial (East Nusatenggara), District (Ngada), and Subdistrict (So'a) Administrations. Excavated materials were shipped to Bandung and are curated in the Geology Museum. CT scans of the fossil materials were conducted in Tokyo, with permission from the Geological Agency, Bandung, Indonesia, and under the supervision of Indonesian research members.
Disturbance	This study did not cause any disturbances.

Reporting for specific materials, systems and methods

We require information from authors about some types of materials, experimental systems and methods used in many studies. Here, indicate whether each material, system or method listed is relevant to your study. If you are not sure if a list item applies to your research, read the appropriate section before selecting a response.

Materials & experimental systems

n/a	Involved in the study
☒	☐ Antibodies
☒	☐ Eukaryotic cell lines
☐	☒ Palaeontology and archaeology
☒	☐ Animals and other organisms
☒	☐ Clinical data
☒	☐ Dual use research of concern
☒	☐ Plants

Methods

n/a	Involved in the study
☒	☐ ChIP-seq
☒	☐ Flow cytometry
☒	☐ MRI-based neuroimaging

Palaeontology and Archaeology

Specimen provenance	The hominin fossils were excavated from Layer II of the Upper Fossil-bearing Interval at Mata Menge, So'a Basin, Flores, Indonesia.
Specimen deposition	The hominin fossils and other fossil specimens are curated at the Geology Museum, Geological Agency, Bandung, Indonesia.
Dating methods	Dates for the reported fossil remains are cited from the previous publications (refs. 5 and 8).

☒ Tick this box to confirm that the raw and calibrated dates are available in the paper or in Supplementary Information.

Ethics oversight	Permission to undertake excavations at Mata Menge was granted by the Indonesian State Ministry of Research and Technology (RISTEK permits 300/SIP/FRP/SM/VIII/2013 and 2183/FRP/SM/X/2015), the provincial government of East Nusa Tenggara in Kupang and the Ngada District Administration.

Note that full information on the approval of the study protocol must also be provided in the manuscript.

Plants

Seed stocks	n/a
Novel plant genotypes	n/a
Authentication	n/a
